# Robot-Assisted Surgery and Multigene Panel Testing for Pheochromocytoma and Paraganglioma Syndrome

**DOI:** 10.7759/cureus.71839

**Published:** 2024-10-19

**Authors:** Kimiaki Takagi, Masaya Hashimoto, Yuka Muramatsu-Maekawa, Keita Nakane, Takuya Koie

**Affiliations:** 1 Urology, Daiyukai General Hospital, Aichi, JPN; 2 Endocrinology, Daiyukai General Hospital, Aichi, JPN; 3 Urology, Gifu University Graduate School of Medicine, Gifu, JPN; 4 Urology, Gifu University, Gifu, JPN

**Keywords:** adrenal pheochromocytoma, dna sequencing-based gene panel testing, hereditary paraganglioma-pheochromocytoma syndrome, men2a, ret gene variant, retroperitoneal paraganglioma, robotic adrenalectomy

## Abstract

Pheochromocytoma and paraganglioma are often associated with hereditary syndromes, particularly those involving genes such as *RET*, which is linked to multiple endocrine neoplasia type 2A. Genetic testing plays a crucial role in diagnosing these conditions, guiding treatment strategies, and providing early intervention options for the affected families. A 57-year-old man presenting with back pain was found to have a left adrenal tumor and a retroperitoneal tumor near the left renal hilum. Hormonal studies and imaging confirmed the diagnosis of pheochromocytoma and paraganglioma. The patient underwent robot-assisted laparoscopic surgery to remove the tumors. Postoperative multigene panel testing identified a mutation in* RET *c.1901G > T (p.Cys634Phe) leading to the diagnosis of multiple endocrine neoplasia type 2A. Despite this genetic finding, no other endocrine tumors, such as medullary thyroid cancer or hyperparathyroidism, were detected at the time of diagnosis. The patient remains under close surveillance for the potential development of associated conditions. This case highlights the importance of comprehensive genetic testing in patients with pheochromocytoma and paraganglioma, particularly when hereditary syndromes are suspected. Genetic insights ensure precise management, allowing for tailored treatment and improved outcomes in patients with hereditary pheochromocytoma and paraganglioma.

## Introduction

Certain forms of pheochromocytoma are hereditary, and several causative genes have been identified. Particularly in cases with suggestive factors, such as an early age of onset, multifocal involvement, or malignancy, there is high suspicion of a hereditary origin. Genetic evaluation is useful for clinical applications such as predicting disease prognosis and guiding early therapeutic interventions for the affected families. Over 20 genes have been implicated in hereditary pheochromocytoma, including *NF1, RET, VHL, SDHB, SDHD, SDHC*, and *SDHAF2* [1]. The *NF1, RET*, and *VHL* genes, which were identified in the 1990s, are associated with neurofibromatosis type 1 (NF1), multiple endocrine neoplasia type 2 (MEN2A), and von Hipple-Lindau (VHL) disease, respectively [2]. *NF1* is primarily a neurocutaneous disorder with benign tumors and skin manifestations. MEN2A affects the endocrine system, with malignant tumors in the thyroid, adrenal, and parathyroid glands. *VHL* involves multiple organ systems, particularly the central nervous system, kidneys, and adrenal glands, with both benign and malignant tumors. The *RET* gene encodes a receptor tyrosine kinase essential for various developmental processes, particularly in the nervous system and kidneys. Its proper function is crucial for normal cellular signaling and development, and mutations in *RET* can lead to severe developmental disorders or predispose individuals to cancer. The *SDHx* genes encode the four subunits of succinate dehydrogenase located in the inner mitochondrial membrane and are considered tumor suppressor genes [3]. Notably, mutations in *SDHB* exhibit a high malignant potential and are expected to serve as prognostic markers. Although genetic testing for pheochromocytoma and paraganglioma (PPGL) is beneficial, it should be conducted selectively based on clinical findings such as family history, age, multifocal and/or extra-adrenal locations, and malignant phenotypes to ensure cost-effectiveness [2]. We report a case of PPGL diagnosed as MEN2A through multigene panel testing following their removal via robot-assisted laparoscopic surgery.

## Case presentation

**Figure 1:**
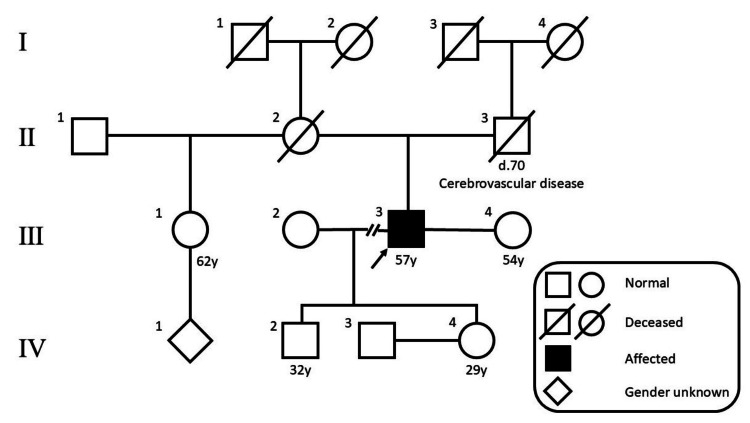
Family tree Patient III was a 57-year-old man. His mother died of unknown reasons, and his father died of subarachnoid hemorrhage at the age of 70.

**Figure 2:**
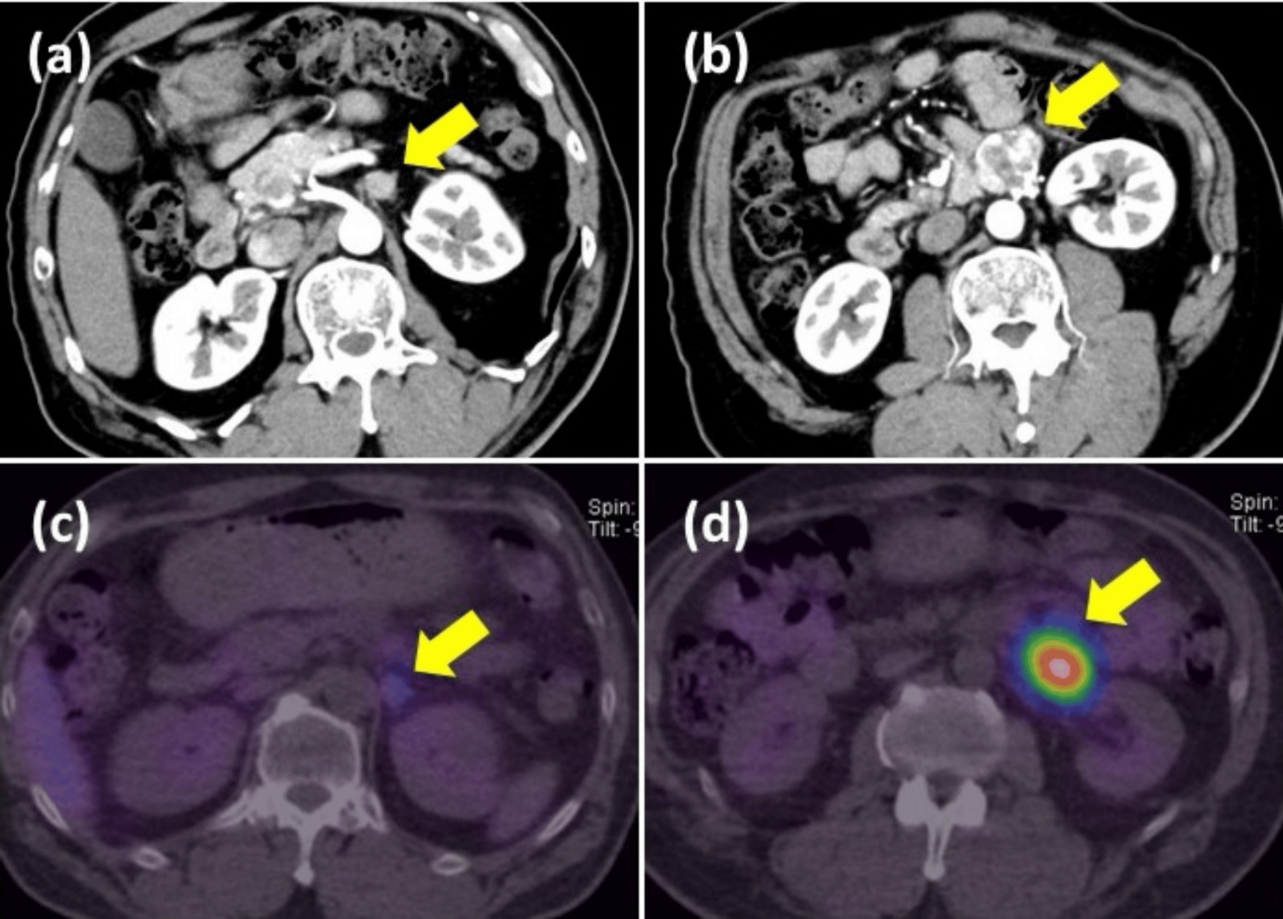
Imaging diagnosis Abdominal enhanced computed tomography revealed a left adrenal tumor (a) and enhanced mass on the left renal hilum (b). Each tumor showed uptake on ^123^I-MIBG scintigraphy (c, d).

A 57-year-old man (Figure 1) presented to the orthopedic department with complaints of back pain. At presentation, his blood pressure was 105/59 mmHg and his heart rate was 75 bpm. He had no hypertension, headaches, sweating, palpitations, or anxiety. Computed tomography revealed a left adrenal tumor and a tumor near the renal hilum (Figure 2). Hormonal studies showed elevated plasma-free metanephrines (132 pg/mL; reference, <130 pg/mL) and free normetanephrines (358 pg/mL; reference, <506 pg/mL) levels. ^123^I-MIBG scintigraphy revealed metaiodobenzylguanidine uptake in both tumors (Figure 2). Based on these findings, the patient was diagnosed as having PPGL.

**Figure 3:**
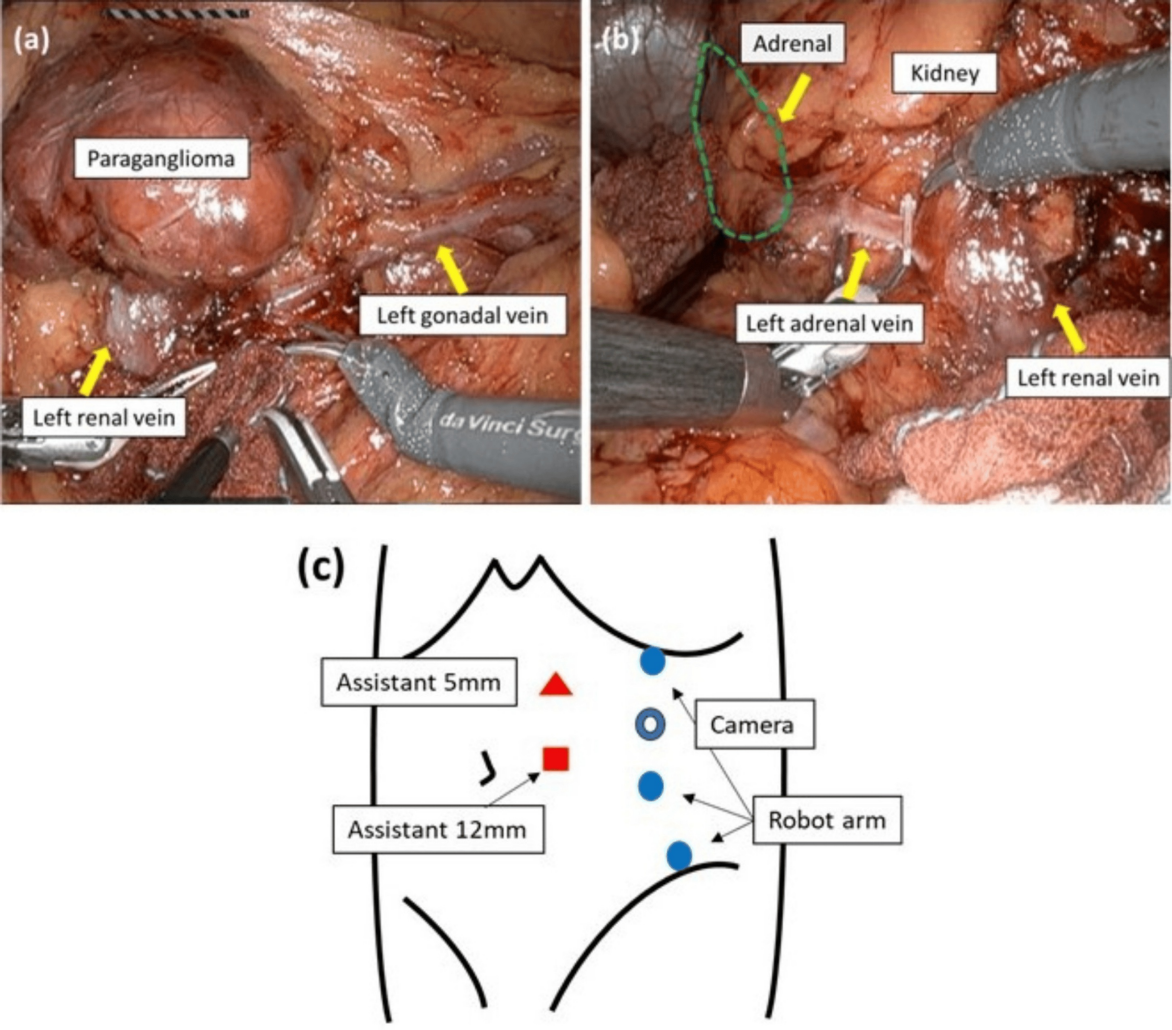
Port placements and intraoperative findings Intraoperative findings during paraganglioma resection (a), adrenal resection (b), and port placement (c)

Robot-assisted laparoscopic surgery was performed with the patient in a right semi-lateral position. Port placements and intraoperative findings are shown in Figure 3. First, the renal hilar tumor was excised. After mobilization of the descending colon, the tumor was identified ventral to the left testicular vein and was removed using a sealing device. Subsequently, a left adrenalectomy was performed. The adrenal artery and vein were cut using a Hem-o-lok^®^. Neither tumor was strongly adherent to the surrounding organs, and both were successfully resected. During adrenal manipulation, blood pressure temporarily increased to 180/90 mmHg, but the administration of phentolamine prevented a hypertensive crisis. The console time was 110 minutes, and the estimated blood loss was 5 mL. No perioperative complications occurred.

**Figure 4:**
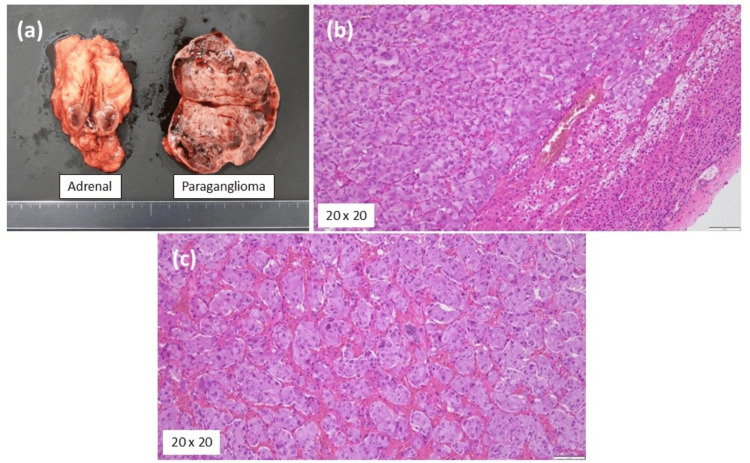
Macroscopic and microscopic findings Macroscopic findings of the tumors (a); Microscopic findings of adrenal tumor (b) and paraganglioma (c) HE, hematoxylin and eosin

The macroscopic and microscopic findings of the adrenal tumor and paraganglioma were similar, with no significant abnormalities or increased mitotic activity noted (Figure 4). Immunohistochemical staining of both tumors was positive for chromogranin A and synaptophysin, consistent with PPGL. The Ki-67 labeling index values were 1.0% and 1.5%, respectively, and the grading system for adrenal pheochromocytoma and paraganglioma was 1. The histopathological examination did not reveal any malignant features.

Following genetic counseling, the patient opted for genetic testing. Multigene panel testing with ACTRisk^TM^ (ACT Genomics, Taipei, Taiwan) (Table 1) identified a heterozygous germline sequence change in *RET *c.1901G > T (p.Cys634Phe). This mutation was interpreted as likely pathogenic according to the American College of Medical Genetics and Genomics guidelines. In the ClinVar database, it was reported as pathogenic (ClinVar entry number: VCV000013911.16). No mutations were found in the *SDH* tumor suppressor genes. The *RET* variant was classified as being associated with MEN2A. Although hyperparathyroidism and thyroid tumors have not been identified in this patient, the penetrance of medullary thyroid cancer in *RET* mutation-positive patients is 100%. Therefore, prophylactic thyroidectomy is being carefully considered.

**Table 1:** The gene list included in the panel ACTRisk^TM^ is a next-generation sequencing assay profiling 67 genes associated with hereditary cancer. For the test, genomic DNA is extracted from the blood sample, and all exon regions of the target gene are analyzed using next-generation sequencing. The resulting report contains evidence-based information regarding variant detection, the risk of developing associated cancers, and disease management, providing those who undergo the test with useful information for early cancer detection and future health management.

ALK	APC	ATM	ATR	AXIN2	BARD1	BLM	BMPR1A
BRCA1	BRCA2	BRIP1	CDH1	CDK4	CDKN2A	CFTR	CHEK2
ENG	EPAS1	EPCAM	FAM175A	FANCC	FH	FLCN	GALNT12
GEN1	GREM1	MAX	MC1R	MDH2	MEN1	MET	MLH1
MRE11	MSH2	MSH3	MSH6	MUTYH	NBN	NF1	NF2
NTHL1	PALB2	PMS2	POLD1	POLE	PRSS1	PTEN	RAD50
RAD51C	RAD51D	RB1	RET	SCG5	SDHA	SDHB	SDHC
SDHD	SDHAF2	SMAD4	SPINK1	STK11	TMEM127	TP53	TSC1
TSC2	VHL	XRCC2					

Three months after surgery, plasma-free metanephrines and free normetanephrines levels had decreased to 22 pg/mL and 112 pg/mL, respectively, and eight months after surgery, they had decreased to below the detection sensitivity (<13 pg/mL and <30 pg/mL). So far, imaging findings have revealed no evidence of metastasis or recurrence. Regular follow-ups, including hormonal and imaging tests, will be conducted in the future.

## Discussion

The primary treatment for PPGL is surgical resection. Robot-assisted laparoscopic surgery is commonly performed in patients with adrenal tumors. Although there have been several reports of robot-assisted surgery for giant adrenal tumors [4-6], there are few reports of robot-assisted surgery for multiple adrenal tumors and paragangliomas. One case report described simultaneous robotic partial cystectomy and right adrenalectomy for hereditary pheochromocytoma-paraganglioma syndrome using dual robotic docking [7]. In our case, the PPGLs were located adjacent to each other on the same side, allowing for single-step surgery without repositioning the patient.

Laparoscopic adrenalectomy is generally associated with less blood loss, shorter hospital stays, and quicker recovery compared to open surgery [8]. In cases where malignancy is a concern, robotic surgery offers advantages such as precise dissection, improved visualization, and reduced complications.

Pathological examination alone cannot distinguish between benign and malignant PPGL. Only after distant metastasis is confirmed can a tumor be clinically diagnosed as malignant or metastatic. Therefore, postoperative follow-up, including endocrinological testing, is recommended. Genetic testing is not universally recommended owing to its cost-effectiveness. However, *SDHB* mutations are found in approximately half of metastatic paragangliomas [9] and may serve as a useful prognostic marker. *SDHB* mutations are reported to correlate with abdominal paraganglioma [10], and multigene panel testing was performed to detect *SDHB *mutations in our patient. Although no *SDHB *mutations were found, a *RET* mutation was identified, leading to the diagnosis of MEN2A. Comprehensive panel testing proved valuable over targeted gene testing limited to *SDHx*.

MEN2A is highly penetrant, with a near 100% lifetime incidence of medullary thyroid cancer in individuals with a *RET* pathogenic variant. Prophylactic total thyroidectomy is recommended for such patients and should be performed by high-volume thyroid surgeons to improve the outcome [11]. We are continuing strict surveillance for medullary thyroid cancer in this patient.

## Conclusions

In conclusion, this case illustrates the successful application of robot-assisted laparoscopic surgery as a single-step procedure for the removal of multifocal pheochromocytoma and paraganglioma. The case emphasizes the importance of comprehensive genetic testing, specifically multigene panel testing, in accurately diagnosing hereditary conditions such as MEN2A. Identifying the RET mutation allowed for targeted management and ongoing surveillance for associated risks, particularly the high likelihood of medullary thyroid cancer. This report advocates for the integration of advanced surgical techniques and genetic evaluation in the clinical management of patients with hereditary pheochromocytoma-paraganglioma syndromes.
